# Genetic Algorithm-Assisted Design of Sandwiched One-Dimensional Photonic Crystals for Efficient Fluorescence Enhancement of 3.18-μm-Thick Layer of the Fluorescent Solution

**DOI:** 10.3390/ma15217803

**Published:** 2022-11-04

**Authors:** Jiantong Song, Guang Feng, Xiao Liu, Haoqiang Hou, Zhihui Chen

**Affiliations:** 1 Key Lab of Advanced Transducer and Intelligent Control System, Ministry of Education and Shanxi Province, Taiyuan University of Technology, Taiyuan 030024, China; 2 College of Physics and Optoelectronics, Taiyuan University of Technology, Taiyuan 030024, China

**Keywords:** one-dimensional photonic crystal, fluorescence enhancement, genetic algorithm

## Abstract

One-dimensional photonic crystal structures have been widely used to enhance fluorescence. However, its fluorescence enhancement is low-fold because of a weak excitation field region. In this paper, we used a genetic algorithm to assist in the design of two photonic crystals based on Al_2_O_3_ and TiO_2_ materials. One of them has a defect consisting of SiO_2_. The Fabry-Perot cavity (FP cavity) formed by the sandwiched photonic crystal achieves up to 14-fold enhancement of the excitation electric field. We modulate the electric field radiation distribution of the fluorescent material by using photonic forbidden bands. For a 3.18 μm thick layer of the fluorescent solution, the structure achieves up to 60-fold fluorescence enhancement. We also discussed that the reason for the different enhancement abilities in different places is the phase change caused by the optical path difference. This design is expected to have applications in display, imaging, etc.

## 1. Introduction

Fluorescence-enhanced structures are widely designed for environmental monitoring, biochemical, medical, and display applications [1,2,3,4,5,6]. Many studies have been conducted to investigate structures for localized fluorescence enhancement, and these studies often use surface plasmas [7,8], resonant cavities [9,10,11,12], and photonic crystals [13,14] to achieve fluorescence enhancement. For example, Boxiang Song et al. [15] designed a structure with a gold sphere as the tip with the excitation electric field localized between the gold spheres; Jungeun Song et al. [16] designed a triangular hole and circular hole array structure with the excitation electric field concentrated in the holes; Nikhil Ganesh et al. [17] designed a perforated photonic crystal with the electric field similarly concentrated in the holes. Songtao Hu et al. [18] coated quantum dots among the gaps of opal structures, which is also the region with the strongest excitation field. However, local area fluorescence enhancement tends to focus on certain hot spots. For large-area fluorescence enhancement, some hot spots will lead to overexcitation of fluorescent material in some regions and low utilization of fluorescent material in other non-hot areas, leading to low overall fluorescence utilization and decreased device lifetime.

Some studies have explored considerable area fluorescence enhancement, e.g., Markus Nyman et al. [19] used surface plasmon as an enhanced excitation field. Weina Zhang et al. [20] also used surface plasmon in combination with microlenses to achieve 260-fold enhancement, but the electric field strength of surface plasmon decays substantially with propagation distance. This means that the layer of fluorescent material cannot be increased in thickness. Hongyun Xuan et al. [21] assembled a one-dimensional photonic crystal with ZnO spheres and coated the quantum dots on the sphere surface, and such a structure obtained a specific excitation electric field enhancement on the sphere surface. Patrick et al. [22] designed a two-dimensional photonic crystal structure to achieve full-plane enhancement, but its enhancement multiplicity varied considerably at different horizontal positions. Ehsan Eftekhar et al. [23] used an opal structure to construct a resonant cavity to achieve fluorescence enhancement of Rhodamine B, but the enhancement multiplicity was not high. Besides, one-dimensional photonic crystals enable large-area fluorescence enhancement. The work of combining photonic crystal and fluorescence enhancement always regards the photonics crystal as a non-loss structure of reflector [24,25,26,27]. Without adding other structures, the one-dimensional photonic crystals own a low fold. As a result, a fluorescence enhancement structure with a large area and high magnification need to be designed. Some works add the fluorescence material as a part of the photonic crystal. But these layers‘ depths are usually lower than one-quarter of the wavelength [28,29,30]. In summary, there are no structures to achieve efficient enhancement in a large area and a high-thickness layer of fluorescent material.

Therefore, we propose to realize fluorescence enhancement using one-dimensional photonic crystals. Since one-dimensional photonic crystals will make the excitation electric field intensity will be precisely the same in one plane, ensuring the same fluorescence excitation enhancement multiplier in the same plane. In addition, we design two photonic crystals with high reflectivity to excitation light and high transmittance to emission light versus high transmittance to excitation light and high reflectivity to emission light through the genetic algorithm-assisted design of photonic crystal parameters and combine the two photonic crystals to achieve an upper limit beyond the excitation enhancement of a single common photonic crystal due to the resonant cavity. Compared to some works with a high Q factor [31,32]. It is worth stating that these designs focus on the source in the cavity. The walls of the optical cavity can own the high reflection for the wavelength of the source, however, our design is aiming for two wavelengths whose dipole source is in the cavity, and the plane wave is out of the cavity. These are the two main processes of photoluminescence. Because the plane wave is out of the cavity, we need to ensure the plane wave passes the back-forward photonic crystal first, which needs a low reflection of the back-forward photonic crystal. As a result, we have a lower Q factor than previously observed.

## 2. Materials and Methods

The finite difference time domain method is one of the effective methods to solve the complex electromagnetic environment generated by the interaction of electromagnetic waves with the matter, and in principle, it can solve the problems of arbitrary forms of electromagnetic fields and electromagnetic wave technology. The finite-time difference method takes a discrete approach to the E and H components of the electromagnetic field with alternate sampling in space and time, and in this way, the Maxwell rotational equations with time variables are transformed into a set of difference equations, and the spatial electromagnetic field is solved step by step in the time axis.

The genetic algorithm [33], as a traditional optimization algorithm, is suitable for problems with a moderate number of optimization parameters. The algorithm filters the excellent set of parameters by the fitness function and generates and retains parameters by cross-variance and genetics. The parameters that we optimize by the algorithm are shown in the Table 1. The fitness function reflects the performance of the corresponding parameter structure (The algorithm flow is shown in Supplementary Section S1: Genetic Algorithm).

Here we use the finite difference time domain method to calculate the excitation light reflectance R_c1p_ of the forward photonic crystal; the transmittance T_c1d_ of the forward photonic crystal point source; the extreme value E_max_ of the square of the enhanced electric field mode generated by the combination of the backward photonic crystal and the forward photonic crystal; and the reflectance R_c2d_ of the back photonic crystal point source. The plane wave approximation represents the value of R_c2d_ (the explanation of this method is given in Supplementary Section S2: Plane wave approximation of spherical waves), and the fitness function is defined by the product of the transmission and reflection values of the corresponding photonic crystal, as shown in Equations (1) and (2). A high fitness_c1_ resulting from the optimization of the genetic algorithm means that the forward photonic crystal has a high reflectance of the excitation light and high transmittance of the emission light, and a high fitness_c2_ means that the backward photonic crystal has high transmittance of the excitation light and high reflectance of the emitted light. Overall, these elements reflect the performance of the structure. The R_c1p_ and E_max_ respond to the properties of the structure excitation process. R_c1p_ is related to forward photonic crystals only; E_max_ is related to forward and back-forward photonic crystals. The R_c2d_ and T_c1d_ respond to the properties of the structure emission process. R_c2d_ is related to back-forward photonic crystals only; T_c1d_ is related to forward photonic crystals only. We calculate different simulation files with a plane wave or dipole, which include one or more parts of the forward PC, backward PC, and layer of the fluorescent solution.

(1) $${\mathrm{fitness}}_{c1}=R_{c1p}\times T_{c1d}$$

(2) $${\mathrm{fitness}}_{c2}=E_{\max}\times R_{c2d}$$

All materials we selected are based on low prices and are easy to access. The Al_2_O_3_ [34], SiO_2_ [35], and TiO_2_ [36] have these characteristics (the index of materials will be shown in the Supplementary Document Section S3: Index of materials in 400–600 nm)

## 3. Results

### 3.1. Photonic Crystal Properties and Performance of the Sandwich Structure

**Figure 1:**
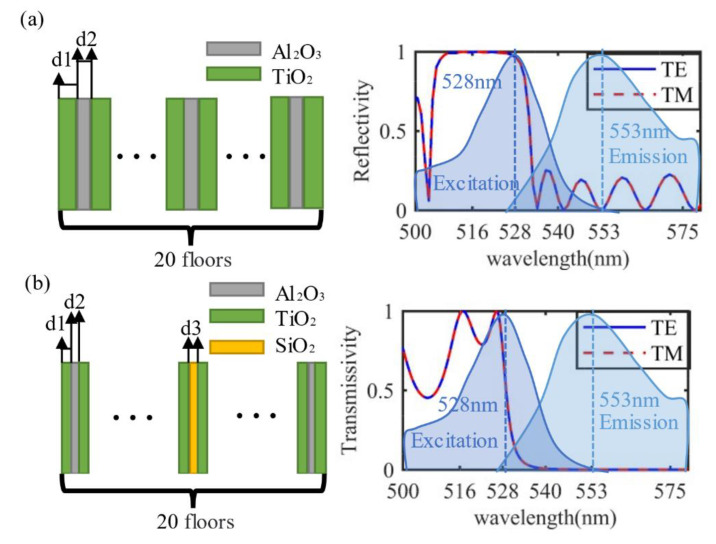
Properties of two photonic crystals for exciting light fields. (**a**) Schematic of the forward photonic crystal (d1 = 150 nm; d2 = 108 nm) and its plane wave reflectivity; (**b**) schematic of the backward photonic crystal (d1 = 81 nm; d2 = 61 nm; d3 = 65 nm) and its plane wave transmittance.

We assume that the layer of the fluorescent solution was about 3.18 um thick, and the refractive index of Rhodamine 6G was nearly 1.593 at 553 nm and 1.4 at 528 nm [37,38]. After the genetic algorithm-assisted design, we finalized the photonic crystal parameters, as shown in Figure 1a,b, and the transmittance and reflectance of the corresponding two photonic crystals for the excitation wavelength vicinity. It can be found that the backward photonic crystal has a high transmittance near the excitation wavelength of 528 nm, while the forward photonic crystal has a high reflectance.

**Figure 2:**
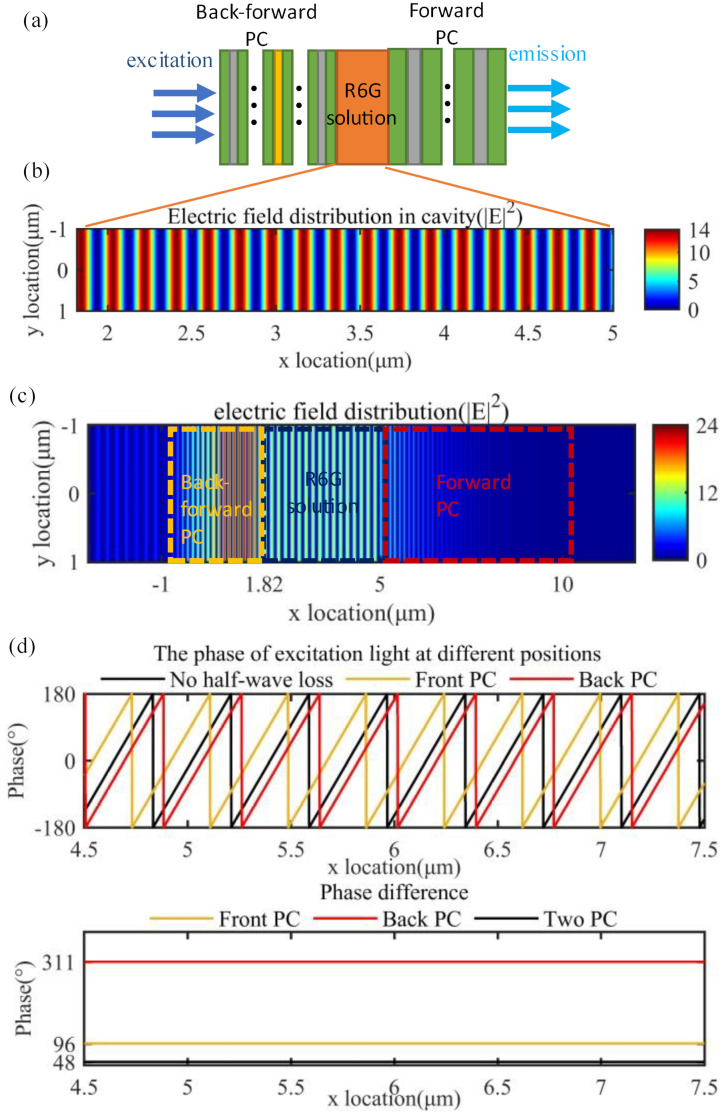
Performance of the structure during excitation. (**a**) Schematic diagram of the structure; (**b**) excitation electric field distribution in the layer of the fluorescent solution (partially enlarged); (**c**) overall electric field distribution at the excitation wavelength; (**d**) phase and phase difference after reflection from photonic crystal.

The overall schematic diagram of the structure is shown in Figure 2a. The excitation light reaches the forward photonic crystal and propagates backward due to the photonic forbidden bands. We indicate the reason for electric enhancement in the layer of the fluorescent solution is the F-P cavity consists of a layer of the fluorescent solution and two photonic crystals. Figure 2d shows that the reflection of the plane wave will cause another change of phase by photonic crystal. The electric field will be enhanced when the phase in the cavity changes by a nearly even number of π (detailed proof is available in Supplementary Document Section S4: F-P cavity). The photonic crystals led to a phase difference of 48 degrees, and when the plane wave propagates in the cavity for one cycle, its optical distance was nearly about 33.7 π. The phase difference caused by the photonic crystal can compensate for the propagating phase difference, which ultimately results in enhanced light field interference. As shown in Figure 2b, the squared value of our electric field mode can reach 10, while it can only reach 4 using a single photonic crystal (see Supplementary Section S5: Quadruple excitation field enhancement generated by a photonic crystal). Its resonant cavity quality factor Q is about 433.567. Figure 2c, on the other hand, shows the electric field distribution as the exciting electric field passes through the structure in a steady state, reaching the forward photonic crystal with almost zero electric field distribution in front of the forward photonic crystal due to its near 100% reflectivity.

**Figure 3:**
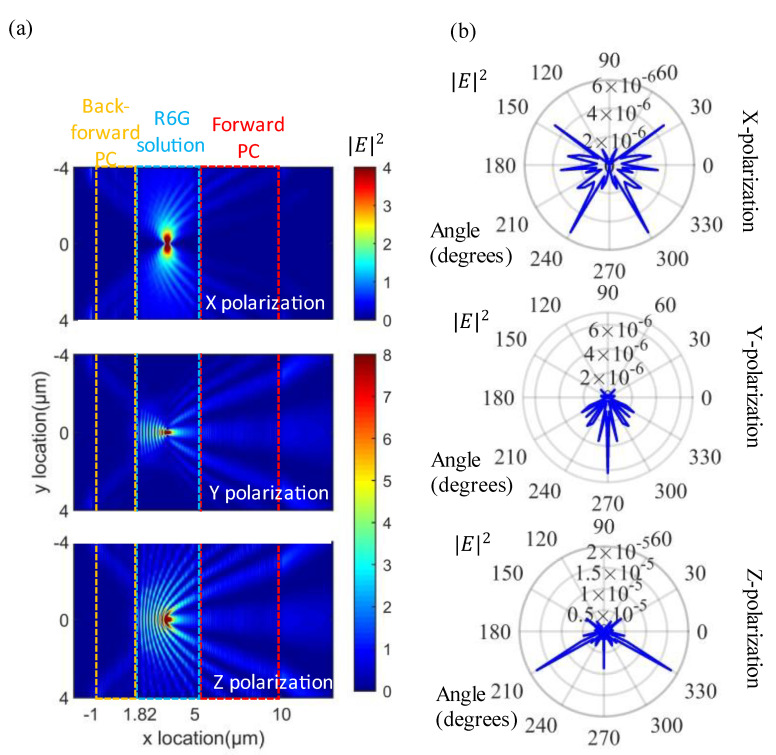
Performance of the structure during emission; (**a**) emitted electric field diagram at three polarizations; (**b**) far-field polar coordinates diagram at three polarizations.

Next, we explored the emission process, as shown in Figure 3a; since the electric dipole has three polarization directions, we calculated the distribution of the electric field mode squared under three polarizations at the center of the layer of the fluorescent solution. We can find that our backward photonic crystal has good reflection regardless of the polarization, and this photonic crystal reflects best at small angles. We tested the far-field radiation map of the structure with the same simulation area size, as shown in Figure 3b. We can find that the structure has good directionality and backward photonic crystal reflection under y and z polarization. Still, the backward photonic crystal had poor reflection under x polarization due to the large difference of electric dipole electric field distribution with y and z polarization distribution modes. But all in all, our structure had some light field directionality during emission.

### 3.2. Effect of the Fluorescent Molecule in Different Positions on Fluorescent Light Enhancement Effect

**Figure 4:**
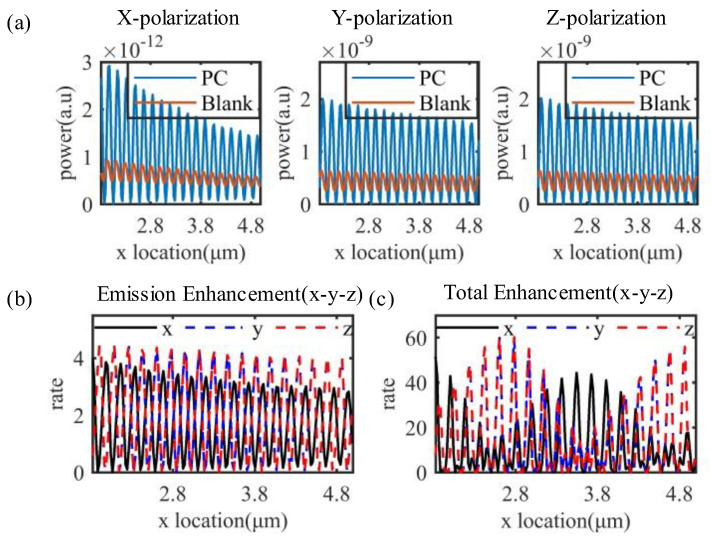
Power value and enhancement multiplier at different positions. (**a**) Power value; (**b**) emission enhancement multiplier; (**c**) overall enhancement multiplier.

Since the field strength distribution of the excitation electric field is periodic in the layer of the fluorescent solution, the span of its electric field amplitude of less than 1 is about 35 nm in the weekly period. Here we tested the enhancement effect of the structure on the fluorescent molecule at different positions. We set the structural unit span of 6um at a distance of 12 um from the fluorescent molecule to set up a monitor of 2 um span to contrast the fluorescence enhancement multiples, as shown in Figure 4a,b, we tested the enhancement multiples of the three polarizations at different positions, respectively. The total enhancement multiplier equation of the structure is shown in Equation (3) [39].

(3) $$R_{\mathrm{total}}=R_{\mathrm{Excitation}}\times R_{\mathrm{Emission}}$$

where R_Excitation_ is the enhancement multiplier of the excitation electric field mode squared, and R_Emission_ is the emission electric field enhancement multiplier. Thus, our structure can achieve about 80% of the fluorescent molecules in the enhancement region.

The final total enhancement multiplier R_total_ versus position is shown in Figure 4c.

**Figure 5:**
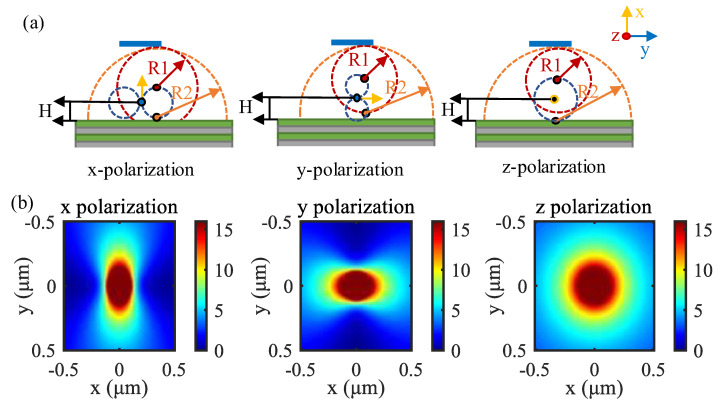
Analysis of the enhancement effect at different positions of fluorescent molecules. (**a**) Schematic diagram of the enhancement effect using the Huygens-Fresnel principle under three polarizations; (**b**) electric field intensity distribution under three polarizations.

Through simulations, we found that the enhancement multiplier of the fluorescent molecule at different positions is not positively correlated with the distance to the backward photonic crystal, but shows a periodic trend (to prove that this phenomenon is not accidental, we verified it in Supplementary Section S6: Effect of reflective structure on the fluorescence enhancement effect at different heights). Here we explain it qualitatively using the Huygens–Fresnel principle, as shown in Figure 5a. Since the distance H of the fluorescent molecule from the backward photonic crystal is constantly changing, the optical field difference between the wave propagating forward to the photonic crystal and the wave propagating forward again after reflection from the backward photonic crystal depends on 2 × H. Therefore, they cancel when the optical range difference corresponds exactly to the phase changeπand increase when the optical range difference is 0. In Figure 4a,b, we can find that the curve changes with a period of about 180 nm, while the half period of the equivalent wavelength of the emitted field among the layer of the fluorescent solution is equal to 173 nm. In addition, the extreme point of the curve under x polarization is different from that under y and z polarization, which is due to the different electric field distribution between x polarization and y and z polarization, as shown in Figure 5b. Therefore, the question of which position will lead to the dominance of phase extinction is somewhat different for x-polarization than for y and z polarization, but the periodicity of their variations is all of half equivalent wavelength. Thus the overall enhancement multiplier for different positions of the structure is shown in Figure 4c, and we achieve the enhancement effect in most of the positions, and the maximum enhancement multiplier can reach 60-fold.

## 4. Conclusions and Discussion

In summary, we designed a one-dimensional photonic crystal large-volume fluorescence enhancement structure containing a Rhodamine 6G (3.18 μm). By adjusting the photonic crystal parameters and considering the introduction of defects through the genetic algorithm-assisted design, the large-volume enhancement of Rhodamine 6G is finally achieved by combining the optical resonant cavity under the action of the forbidden band and the conduction band of the photonic crystal. Through numerical calculations, we can achieve a maximum of 60-fold enhancement under x, y, and z polarizations. We analyzed the enhancement conditions of the excitation electric field in the FP cavity. We also illustrate the periodic enhancement characteristics of the reflective fluorescence enhancement structure at large volume enhanced fluorescence and perform a qualitative analysis. This design can be used in fields such as displays and further improve device lifetime.

## Figures and Tables

**Table 1:** Parameters for genetic algorithm optimization.

Parameter	Description
d_TiO2_f_	The thickness of TiO_2_ for forward photonic crystal
d_Al2O3_f_	The thickness of Al_2_O_3_ for forward photonic crystal
d_SiO2_f_	The thickness of SiO_2_ for forward photonic crystal
defect_f	Forward photonic crystal with defects (Yes or No)
d_TiO2_b_	The thickness of TiO_2_ for back-forward photonic crystal
d_Al2O3_b_	The thickness of Al_2_O_3_ for back-forward photonic crystal
d_SiO2_b_	The thickness of SiO_2_ for back-forward photonic crystal
defect_b	Back-forward photonic crystal with defects (Yes or No)

## Data Availability

The data presented in this study are available in Supplementary Materials.

## Supplementary Materials

The following supporting information can be downloaded at: https://www.mdpi.com/article/10.3390/ma15217803/s1. Ref. [40] was included in Supplemental File.
