# Supplementary material for: Genetic Algorithm-Assisted Design of Sandwiched One-Dimensional Photonic Crystals for Efficient Fluorescence Enhancement of 3.18-μm-Thick Layer of the Fluorescent Solution
Source: Materials (Basel). 2022 Nov 4;15(21):7803. doi: 10.3390/ma15217803 (PMC9658013; doi:10.3390/ma15217803)
Supplement: Supplementary file 1 [file materials-15-07803-s001.zip › materials-1946818-supplementary.pdf]

Genetic Algorithm-assisted Design of Sandwiched One-dimensional Photonic Crystals for Efficient Fluorescence Enhancement of 3.18-μm-thick Layer of the Fluorescent Solution: supplement

Jiantong Song[1,2], Guang Feng [1,2], Xiao Liu [1,2] , Haoqiang Hou[1,2]and Zhihui Chen[1,2*]

[1] Key Lab of Advanced Transducer and Intelligent Control System, Ministry of Education and Shanxi Province, Taiyuan University of Technology, Taiyuan 030024, China;

[2] College of Physics and Optoelectronics, Taiyuan University of Technology, Taiyuan 030024, China

*Corresponding author: huixu@ 126.com;

Genetic Algorithm-assisted Design of Sandwiched One-dimensional Photonic Crystals for Efficient Fluorescence Enhancement of 3.18-μm-thick Layer of the Fluorescent Solution: supplemental document

List of contents:

Section S1 –Genetic Algorithm
Section S2 –Plane wave approximation of spherical waves
Section S3 –Index of materials in 400-600nm
Section S4 –F-P cavity
Section S5 –Quadruple excitation field enhancement generated by a photonic crystal
Section S6 –Effect of reflective structure on the fluorescence enhancement effect at different heights
References

Section S1 –Genetic Algorithm

Genetic algorithms, as traditional optimization algorithms, are a good choice when targeting structures with few parameters. Figure 1 is the flowchart of our algorithm.

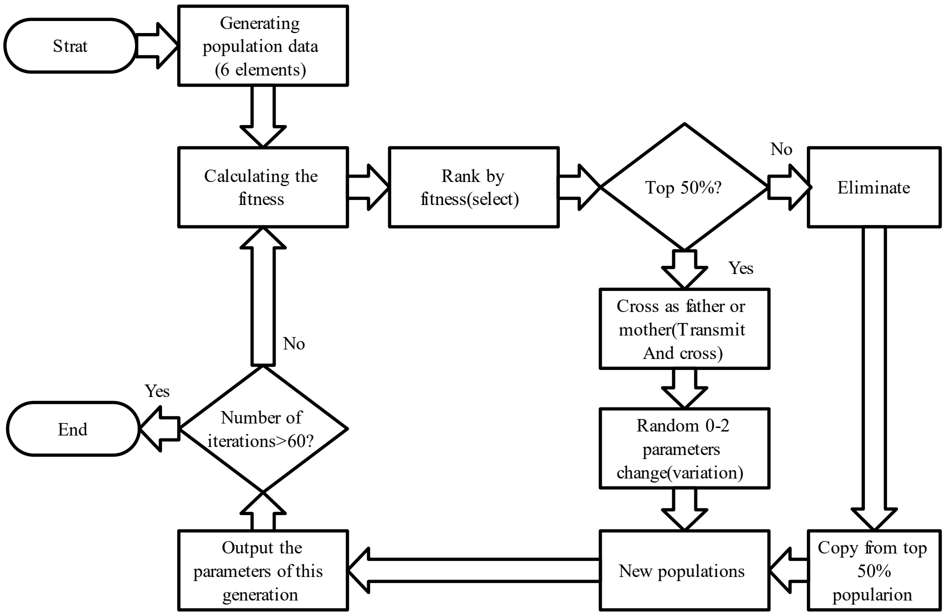

Figure S1. Flowchart of Genetic Algorithm.

Section S2 –Plane wave approximation of spherical waves

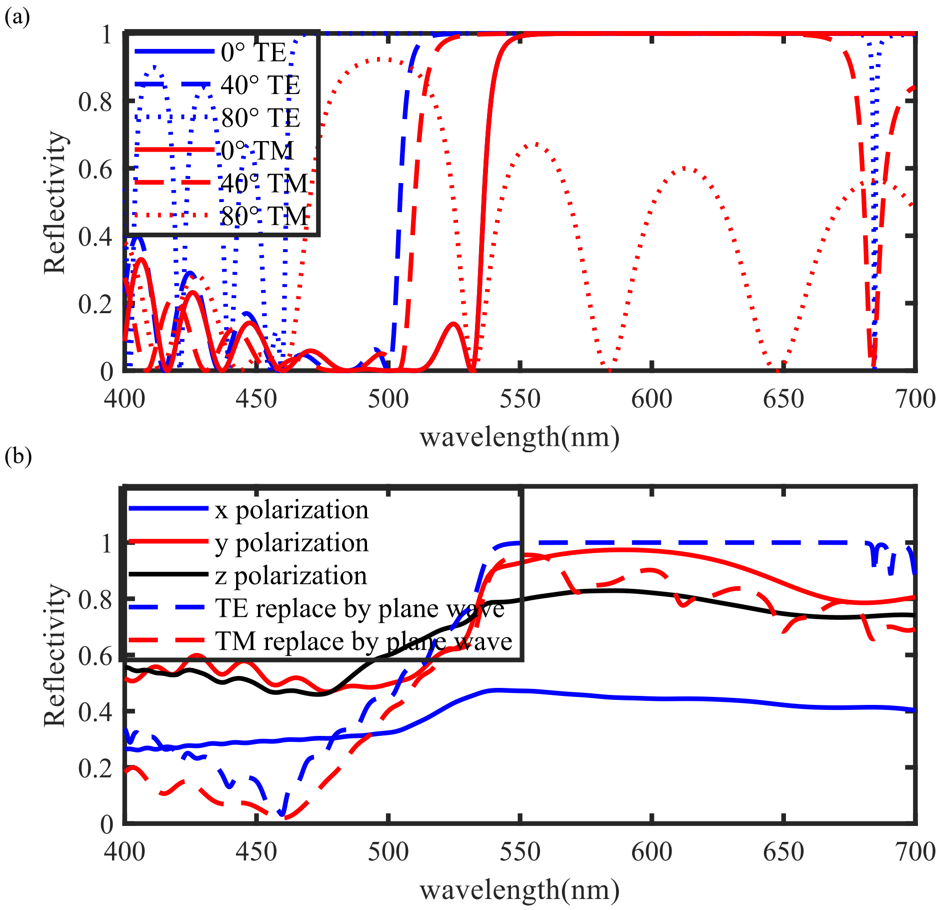

Figure S2. Plane-wave approximation of spherical wave: (a) reflectivity curves of 0-80 degree plane wave incident photonic crystal, (b) reflectivity curves of electric dipole incident photonic crystal with three polarizations, and reflectivity curves using plane wave approximation.

We use the transmission matrix method for 300-700 nm plane waves incident at 0-80 degrees to a double-layer photonic crystal with refractive indices 1 and 2, as shown in Figure 2(a). Meanwhile, we use the time-domain finite difference method for the reflection curves of electric dipoles with different polarizations located above the photonic crystal, as shown in Fig. 2(b). After averaging the transmission matrix method, we can find that the reflection effect of electric dipoles can be better approximated using this plane wave approximation. Also, in the case of plane waves, the reflection peaks are shifted toward lower wavelengths as the angle increases, So we further simplified the representative electric dipole approach by focusing on plane waves to excite wavelengths and longer wavelengths instead of electric dipoles in the case of vertically incident photonic crystals.

Section S3 –Index of materials in 400-600nm

	Al2O3	
Wavelength(μm)	Re(index)	Im(index)
4	1.674	0
4.167	1.667	0

4.348	1.658	0
4.545	1.647	0
4.762	1.636	0
5	1.624	0
5.263	1.607	0
5.556	1.624	0.002
5.882	1.6	0.002

SiO2		
Wavelength(μm)	Re(index)	Im(index)
4.167	1.383	0.000107
4.545	1.365	0.000256
5	1.342	0.00398
5.556	1.306	0.00563
5.882	1.278	0.00594

TiO2		
Wavelength(μm)	Re(index)	Im(index)
0.4	2.337928	0
0.41	2.314541	0
0.42	2.294437	0
0.43	2.276919	0
0.44	2.261491	0
0.45	2.247783	0
0.46	2.235515	0
0.47	2.224466	0
0.48	2.214461	0
0.49	2.205359	0
0.5	2.197043	0
0.51	2.189417	0
0.52	2.182399	0
0.53	2.17592	0
0.54	2.169924	0
0.55	2.164358	0
0.56	2.15918	0
0.57	2.154353	0
0.58	2.149843	0
0.59	2.145621	0
0.6	2.141661	0

Section S4 – F-P cavity

The principle of FP cavity can be explained as multi-beam interference[1]. When the phase difference of light is below 90 degrees, the light field interferes and the amplitude increases.

We assume that the expression of the plane wave incident into the left side of the layer of fluorescent solution is

$$E_1 = Acos(\omega t + \varphi_0)$$

Where φ0 is the initial Phase, ω is the electromagnetic wave frequency. A is the initial amplitude of the plane wave.

When the plane wave has been reflected once towards the front photonic crystal.

$$E_2 = R_1 \times Acos(\omega t + \varphi_0 + \Delta\varphi + \varphi_1)$$

Where △φ is the phase change caused by a plane wave propagating from one side of the cavity to the other. R1 is the reflectance of the forward photonic crystal. φ1 is the phase change caused by reflection through the forward photonic crystal.

When the plane wave has been reflected once towards the back photonic crystal.

$$E_4 = R_1 \times R_2 \times Acos(\omega t + \varphi_0 + \varphi_1 + 2 \times \Delta\varphi + \varphi_2)$$

Where φ2 is the phase change caused by reflection through the back photonic crystal. R2 is the reflectance of the back photonic crystal.

When the plane wave reaches the front photonic crystal again.

$$E_5 = R_1 \times R_1 \times R_2 \times Acos(\omega t + \varphi_0 + 2 \times \varphi_1 + 3 \times \Delta\varphi + \varphi_2)$$

We can find that the actual phase difference is $\varphi_1 + 2 \times \Delta\varphi + \varphi_2$. If $\varphi_1 + 2 \times \Delta\varphi + \varphi_2 = 2n\pi$. (n is an integer). Interference enhancement of the optical field will be maximized. The phase change is shown in figure3.

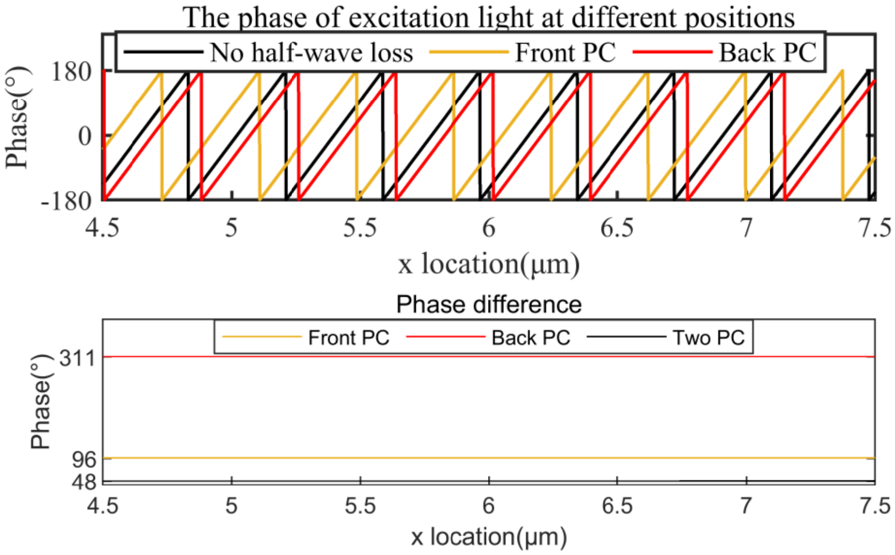

Figure S3. Phase and phase change at different positions

Section S5 –Quadruple excitation field enhancement generated by a photonic crystal

Here we design for 500 nm wavelength. Since the excitation light is a plane wave, we can ensure 100% reflectivity at 500 nm wavelength according to Eq. 1, where n denotes the refractive index of the photonic crystal layer, d is the thickness of the photonic crystal layer, and λ is the incident wavelength.

$$n \times d = \frac{\lambda}{4} \tag{1}$$

The final structure is shown in Fig. 4(a), and the reflectivity curve of the system is shown in Fig. 4(b), where the reflectivity is 100% near 500 nm. The electric field mode squared maximum value of 4 can be seen from the electric field diagram in Fig. 4(c).

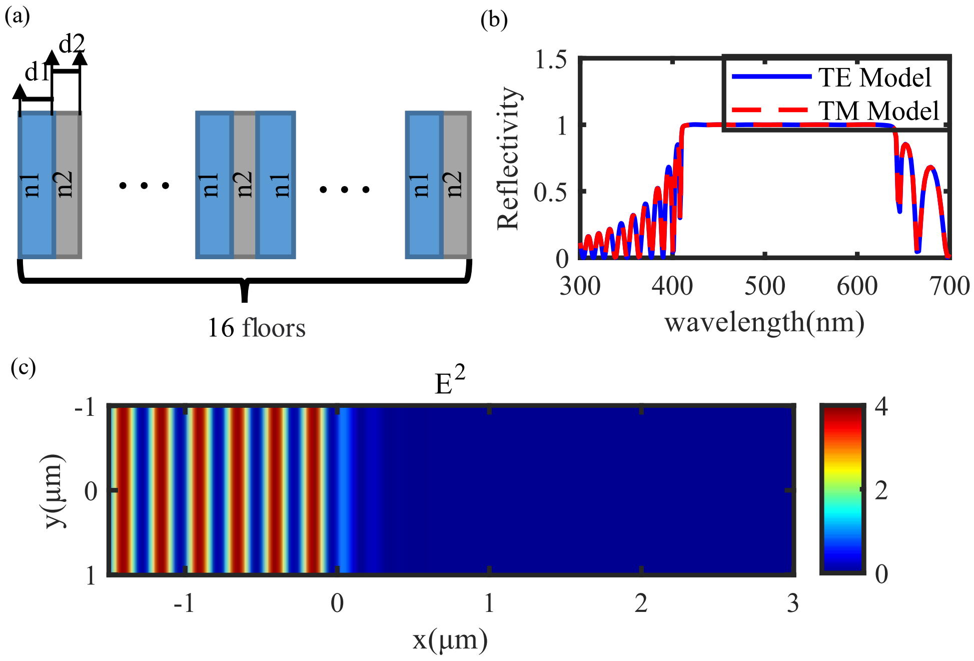

Figure S4. A photonic crystal property: (a) photonic crystal structure designed for 500 nm plane wave (n1=1,d1=125 nm,n2=2,d2=62.5 nm), (b) reflection spectrum of photonic crystal, (c) electric field distribution at 500 nm wavelength.

Section S6 –Effect of reflective structure on the fluorescence enhancement effect at different heights

To demonstrate that the periodically varying effect in the article is not unique to photonic crystals, we take a one-directional boundary condition as the metal boundary condition, which can be considered a total reflection plane. As shown in Figure 5, we also see the effect of periodic variation of the monitor receiving power value.

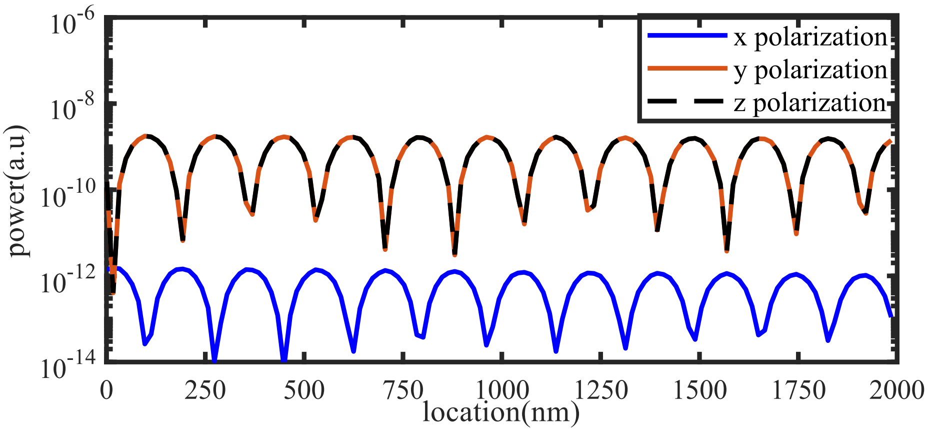

Figure S5. The effect of different positions of power value of the reflective structure.

References

[1] Min, C.; Fuli, Z.; Jianwen, D. OPTICS, August 2018. pp. 88–133.
